# Comparison of the analytical performance of the Oncomine dx target test focusing on bronchoscopic biopsy forceps size in non‐small cell lung cancer

**DOI:** 10.1111/1759-7714.14411

**Published:** 2022-04-05

**Authors:** Tadashi Sakaguchi, Yoichi Nishii, Akemi Iketani, Seiya Esumi, Maki Esumi, Kazuki Furuhashi, Yuki Nakamura, Yuta Suzuki, Kentaro Ito, Kentaro Fujiwara, Koji Katsuta, Osamu Taguchi, Osamu Hataji

**Affiliations:** ^1^ Matsusaka Municipal Hospital, Respiratory Center Matsusaka Mie Japan; ^2^ Matsusaka Municipal Hospital, Pathology Department Matsusaka Mie Japan

**Keywords:** endobronchial biopsy, next‐generation sequencing, non‐small cell lung cancer, Oncomine dx target test, transbronchial biopsy

## Abstract

**Background:**

Next‐generation sequencing (NGS) has been implemented in clinical oncology to analyze multiple genes and to guide targeted therapy. Although the pathological diagnosis and biomarker tests for patients with advanced lung cancer have mostly been obtained with small biopsy samples, especially with bronchoscopic approaches, the performance for NGS with respect to the different sizes of biopsy forceps remains little known.

**Methods:**

We retrospectively reviewed consecutive patients with non‐small cell lung cancer, whose FFPE samples were obtained by endobronchial biopsy/transbronchial biopsy and were submitted for the Oncomine Dx Target Test (ODxTT). We compared the analytical performance for ODxTT with respect to the size of biopsy forceps.

**Results:**

A total of 103 samples were identified. The success rate of the ODxTT for the group with all samples obtained with small forceps biopsies (70%) was lower than that of the group with some or all samples obtained with standard forceps biopsies (83%), although without a statistically significant difference (*p* = 0.20). With regard to the reason for unsuccessful analysis, the proportion of the samples which did not pass the nucleic acid concentration threshold in the former group (15%) was higher compared with that of the latter group (4%) (*p* = 0.08). The proportion of tissue size 4 mm^2^ or larger in the former group (70%) was lower than that in the latter group (93%) (*p* = 0.01).

**Conclusion:**

The analysis of ODxTT for specimens biopsied using only small forceps is prone to be unsuccessful due to an insufficient amount of nucleic acid.

## Graphical abstract

Among a total of 103 samples obtained by endobronchial biopsy/transbronchial biopsy, the success rate of the ODxTT for the group with all samples obtained with small forceps biopsies (70%) was lower than that of the group with some or all samples obtained with standard forceps biopsies (83%). As for the reason for unsuccessful analysis, the proportion of the samples which did not pass the nucleic acid concentration threshold in the former group (15%) was higher compared with that of the latter group (4%).

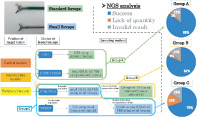

## INTRODUCTION

Targeted therapies for advanced non‐small cell lung cancer (NSCLC) patients harboring driver oncogene alterations have been proven to have promising antitumor activities, and are generally recommended as the first‐line therapy for these patients in clinical guidelines.^1^, ^2^, ^3^ Conventionally, single‐gene tests were conducted as companion diagnostics to select suitable patients for targeted therapies. However, as the number of driver oncogene alterations recommended for detection in clinical settings has increased, it has become increasingly difficult to conduct all of the multiple single‐gene tests due to increased tissue consumption. Furthermore, the success rates of the ordered tests have decreased.^4^

Next‐generation sequencing (NGS) can detect multiple gene variants simultaneously, enabling comprehensive genetic testing. The Oncomine Dx Target Test (ODxTT) is one of the NGS panels, and was approved by the US Food and Drug Administration in June 2017.^5^ Since February 2019 this test has been approved by the Ministry of Health, Labor and Welfare of Japan as a companion diagnostic for targeted therapies on four driver mutations: epidermal growth factor receptor (EGFR), anaplastic lymphoma kinase (ALK), ROS proto‐oncogene 1, receptor tyrosine kinase (ROS1), and B‐Raf proto‐oncogene, serine/threonine kinase (BRAF) (p.V600E). Furthermore, RET fusions have been added as a companion diagnostic of the ODxTT since September 2021 in Japan.

For patients with advanced lung cancer, the pathological diagnosis and biomarker tests have mostly been obtained with small biopsy samples, such as endobronchial biopsy/transbronchial biopsy (EBB/TBB), endobronchial ultrasound‐guided transbronchial needle aspiration (EBUS‐TBNA) and CT‐guided needle biopsy (CTNB). Although the quantity of tumor cells in these biopsy samples is smaller than in surgical samples, some reports have shown the good feasibility of NGS panel testing using small biopsy samples.^6^, ^7^, ^8^ Standard forceps biopsy is generally recommended to obtain enough sample material for NGS analysis compared with small forceps biopsy for EBB/TBB; however, the comparison of analytical performance for NGS based on the different size of biopsy forceps on EBB/TBB remains little known.

Therefore, in this study we retrospectively evaluated the analytical performance of the ODxTT on EBB/TBB samples focusing on the biopsy forceps size in clinical settings.

## METHODS

### Patient selection

This retrospective study was conducted at Matsusaka Municipal Hospital, Japan. We reviewed electronic data from consecutive patients who were diagnosed with NSCLC and whose formalin‐fixed and paraffin‐embedded (FFPE) samples obtained by EBB/TBB had been submitted for the Oncomine Dx Target Test (ODxTT) (Ion Torrent PGM Dx Sequencer; Thermo Fisher Scientific) from August 2019 to July 2020. Samples collected in other hospitals, and archived samples, were excluded. Clinical data assessments included: patient characteristics, CT findings, sampling methods, pathological findings, and the results of genetic tests. This study was approved by the institutional review board of Matsusaka Municipal Hospital (IRB number J‐76‐200 410‐5‐2). Informed consent was obtained by the opt‐out method.

### Sampling methods

**FIGURE 1:**
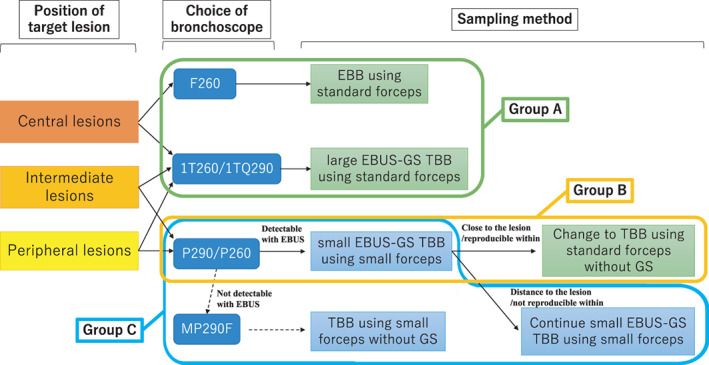
Strategy of sampling methods and grouping focused on usage of forceps size. Group A circled in green is the group performed EBB/TBB using only standard forceps; group B circled in orange is the group performed TBB using standard forceps and small forceps; group C circled in blue is the group performed TBB using only small forceps. Abbreviations: EBB, endobronchial biopsy; EBUS, endobronchial ultrasonography; GS, guide sheath; TBB, transbronchial biopsy

Virtual bronchoscopic navigation (VBN) was used in combination with CT, X‐ray fluoroscopy, and radial‐probe endobronchial ultrasonography with a guide sheath (EBUS‐GS) (K‐203: large guide sheath kit, or K‐201: small guide sheath kit; Olympus). We used 1.9 mm outside diameter standard forceps (FB‐231D, Olympus) and 1.5 mm outside diameter small forceps (FB‐233D, Olympus). We used one of the following bronchoscopes: BF‐1TQ290, BF‐1 T260, BF‐P290, BF‐P260, BF‐F260 or BF‐MP290F (Olympus Medical Systems). The general strategy in our institution regarding the choice of bronchoscope and sampling method is shown in Figure 1. The lesion locations were classified as being in the central, intermediate, or peripheral one‐third of the CT lung field, as classified by Baaklini et al.^9^ When the targets were directly detectable endobronchial lesions, we selected a BF‐F260 scope and performed EBB using standard forceps from the magenta area indicated by autofluorescence imaging. When the targets were central or intermediate lesions, and easy to approach using the large EBUS‐GS method, we selected either a BF‐1TQ290 or BF‐1 T260 scope, and performed TBB using standard forceps with a large EBUS‐GS (large EBUS‐GS TBB). When the targets were intermediate or peripheral lesions, and difficult to approach with the large EBUS‐GS method, we selected either a BF‐P260 or BF‐P290 scope and performed TBB using small forceps with a small EBUS‐GS (small EBUS‐GS TBB). After performing small EBUS‐GS TBB about five times, if we could proceed with the tip of the scope close to the target lesion, and could reproducibly identify areas within the lesions using EBUS, we additionally performed TBB using standard forceps without GS. Meanwhile, if we could not proceed with the tip of scope close to the lesion, and when it was difficult to identify areas within the lesions using EBUS reproducibly, we continued using the small EBUS‐GS TBB about five more times. When the targets were difficult to detect using the small EBUS‐GS method, we changed the scope to a BF‐MP290F, which is an ultrathin bronchoscope, whose forceps channel was not applicable to small EBUS‐GS or standard forceps, and performed TBB using small forceps.

### Sample processing and genetic tests

Small tissue samples collected by EBB/TBB were immediately placed in 10% neutral buffered formalin (NBF) and fixed over about 12 to 24 h at room temperature. Formalin‐fixed tissues underwent serial processing and were then embedded in paraffin to create FFPE blocks. Both the number of tumor cells, and the tumor content of the sample stained with hematoxylin and eosin, were evaluated by skilled cytopathologists. In some samples obtained in 2020, macro‐dissection was performed as needed in our institution. Multiple samples with suitable tumor content were selected with marking and macro‐dissection, collectively placed on a slide, and submitted for the ODxTT. If the tumor content was <20% after marking and macro‐dissection in small biopsy samples, or the amount of tumor cells was insufficient, the sample was not submitted for the ODxTT. For the ODxTT, 10 to 20 slide‐mounted 5–10‐μm sections of small biopsy samples, depending on each sample volume, were submitted to LSI Medience Laboratories (Tokyo, Japan). LSI Medience Laboratories performed the ODxTTs based on Thermo Fisher's Ion AmpliSeq technology.^5^

### Outcomes

We evaluated the success rate for the ODxTT and tissue size measured in area, dividing cases into three groups as shown in Figure 1 (group A is the group with EBB/TBB performed using only standard forceps, circled in green; group B is the group with TBB performed using standard forceps and small forceps, circled in orange; group C is the group with TBB performed using only small forceps, circled in blue.). The main analysis is a comparison of the success rate for the ODxTT and tissue size between the groups containing the standard forceps biopsy samples, and the group containing only small forceps biopsy samples (combined groups A and B vs. group C). A subanalysis is a comparison between the groups containing only small forceps biopsy samples (group B vs. group C). Results of the ODxTT were considered successful if all results were valid for the four‐companion diagnostic genetic targets (EGFR, ALK, ROS1, and BRAF) approved in Japan during the period covered. Conversely, the results were regarded as unsuccessful if the sample did not pass the nucleic acid concentration threshold, or if one or more of the genetic target results mentioned above were invalid due to a failure to meet the DNA or RNA sample quality control (QC) metrics, or no call.

**FIGURE 2:**
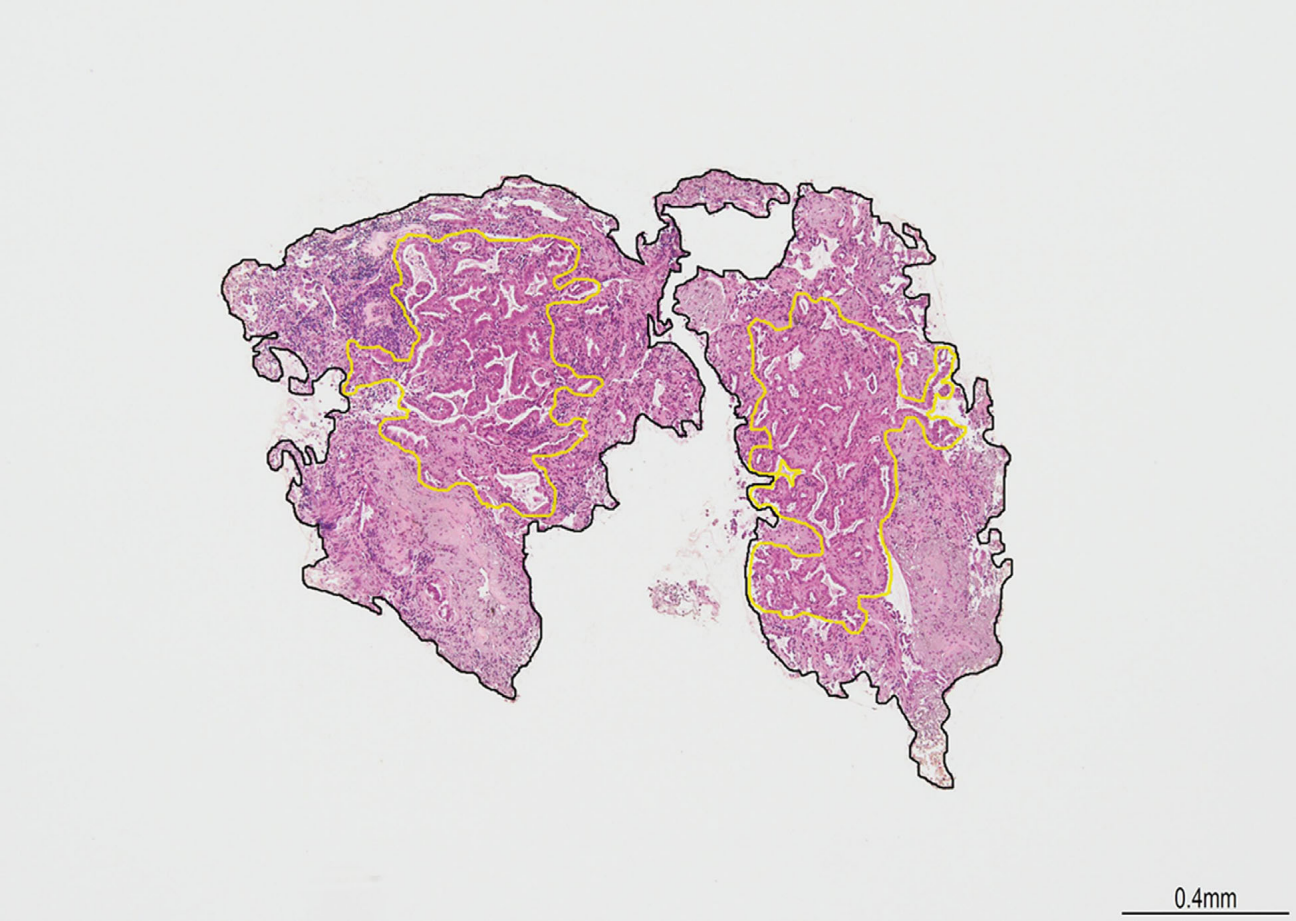
Evaluation of the tissue and tumor size. Areas surrounded by black are tissue size. Areas surrounded by yellow are tumor size

In order to evaluate the tissue size, we measured the histological sample area using microscope camera control unit software (DS‐L4, Nikon Corporation) for each group. The areas were measured by T.S. and A.I. under the supervision of an experienced pathologist (K.K.) in a blinded situation for the categorized group. An example is shown in Figure 2. We compared the tissue size, defined as the sum of sample areas including only the samples containing tumor cells for each case, and assessed the proportion of the total area of 4 mm^2^ or larger, which was reported as a favorable tissue size for the success of ODxTT analysis.^7^

### Statistical analysis

Statistical analyses were performed using Student's *t*‐test and Fisher's exact test for continuous and categorical variables. Statistical analyses were performed using SPSS software, version 26.0 (SPSS Inc.). *p*‐values of less than 0.05 were considered statistically significant.

## RESULTS

### Sample characteristics

A total of 103 samples were identified for comparison analysis. The sample characteristics among the three groups are shown in Table 1. Among the 103 samples, 33 (32%) samples were classified as group A, 50 (49%) samples were classified as group B, and 20 (19%) samples were classified as group C. There was some variability in the sample characteristics between the three groups because the sampling method and bronchoscope type were decided upon depending on the location of the lesions and accessibility as mentioned above. Most of the target lesions in groups B and C were intermediate and peripheral lesions, while those of group A were proximal lesions. The median diameter of the lesions in group A was greater than that in groups B or C. More pure ground‐glass nodules and part solid nodules were contained in group C compared with groups A and B.

**TABLE 1:** Sample characteristics

	Group A		Group B		Group C	
	*n* = 33	(%)	*n* = 50	(%)	*n* = 20	(%)
Median age	76		74		75	
Range	55–93		55–90		39–94	
Sex						
Female	9	27	18	36	4	20
Histology						
Nonsquamous	20	61	33	66	15	75
Squamous	13	39	17	34	5	25
Radiological location						
Central	25	76	8	16	2	10
Intermediate	6	18	21	42	9	45
Peripheral	2	6	21	42	9	45
Median lesion size (mm)	41		27		25	
Range	11–110		7–67		9–52	
Nodule classification						
Pure GGN	1	3	0	0	2	10
Part solid nodule	1	3	6	12	4	20
Solid nodule	31	94	44	88	14	70
CT bronchus sign						
Positive	32	97	50	100	19	95

Abbreviations: GGN, ground‐glass nodule.

### Success rate of the ODxTT

The results for the ODxTT for each group are shown in Table 2. The median number of samples collected were comparable in the three groups; Group A: nine times (range [2–12]), group B: 10 times (range [6–15]), group C: 8.5 times (range [3–12]). The success rate of the ODxTT in group C (70%) was lower than that in the combined groups A and B (83% [group A: 85%, group B: 82%]) (*p* = 0.20). With regard to the reason for unsuccessful analysis, the proportion of the sample which did not pass the nucleic acid concentration threshold in group C (15%) was higher than in the combined groups A and B (4% [group A: 3%, group B: 4%]) (*p* = 0.08), although both were not statistically significant. The success rate of the ODxTT in group B was higher than in group C, and the proportion of unsuccessful analysis due to not passing the nucleic acid concentration threshold in group B was lower than in group C, although both were also not significantly different (*p* = 0.33 and *p* = 0.13). The proportion of unsuccessful results due to invalid DNA and RNA analysis were comparable in all three groups.

**TABLE 2:** Analysis results of OD × TT

	Group A		Group B		Group C	
	*n* = 33	(%)	*n* = 50	(%)	*n* = 20	(%)
Results of OD × TT						
Success of analysis	28	85	41	82	14	70
Not passing the nucleic acidconcentration threshold	1	3	2	4	3	15
Invalid results for DNA only(*EGFR, BRAF*)	3	9	4	8	2	10
Invalid results for RNA only(*ALK, ROS1*)	1	3	3	6	1	5
Invalid results for DNA and RNA	0	0	0	0	0	0

Abbreviations: ALK, anaplastic lymphoma kinase; BRAF, B‐Raf proto‐oncogene, serine/threonine kinase; EGFR, epidermal growth factor receptor; ODxTT, Oncomine Dx Target Test; ROS1, ROS proto‐oncogene 1, receptor tyrosine kinase.

### Tissue and tumor size

**FIGURE 3:**
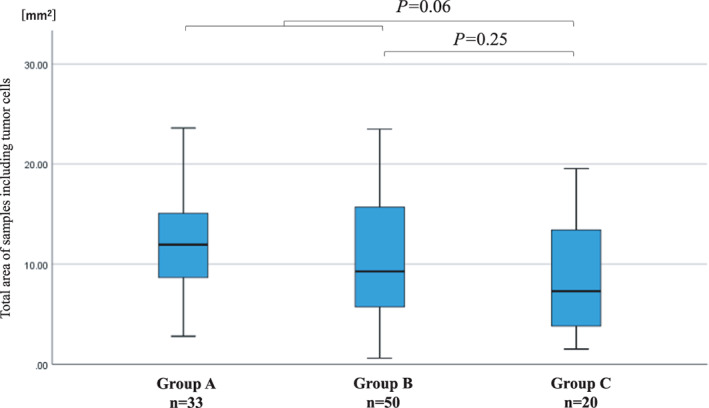
The comparison of tissue size. The tissue size was evaluated as the sum of sample areas, including only the samples containing tumor cells for each case. *p*‐values of less than 0.05 were considered statistically significant

The tissue size comparison is shown in Figure 3. The median tissue size was 11.9 mm^2^ in group A, 9.2 mm^2^ in group B, and 7.3 mm^2^ in group C. The tissue size of the combined groups A and B was larger than that of group C, although not statistically significant (*p* = 0.06). Additionally, the comparison between groups B and C was also not statistically significant (*p* = 0.25). As shown in Table 3, the proportion of tissue size of 4 mm^2^ or larger in group C (70%) was significantly lower than that of the combined groups A and B (93%) (*p* = 0.01). Additionally, the proportion of tissue size of 4 mm^2^ or larger in group C was lower than that of group B (90%), although not statistically significant (*p* = 0.06).

**TABLE 3:** Total area of samples including tumor cells related to 4 mm^2^ cutoff

Total area of samplesincluding tumor cells	Group A		Group B		Group C	
	*n* = 33	(%)	*n* = 50	(%)	*n* = 20	(%)
≥4 mm^2^	32	97	45	90	14	70
<4 mm^2^	1	3	5	10	6	30

Furthermore, we performed several additional evaluations as exploratory analyses. First, we measured tumor size as shown in Figure 2, and compared the values between the groups as was done both in the main analysis (combined groups A and B vs. group C) and in the subanalysis (groups B vs. C). The median tumor size was 3.6 mm^2^ in group A, 2.0 mm^2^ in group B, and 1.5 mm^2^ in group C. The tissue size of the combined groups A and B was significantly larger than that of group C (*p* < 0.01); however, the comparison between groups B and C was not statistically significant (*p* = 0.14) (Figure S1). Second, to evaluate the usefulness of adding TBB using standard forceps without GS to small EBUS‐GS TBB in group B, we compared the proportion of sample specimens that contained tumor cells per biopsy between each forceps among group B. In group B, the total number of biopsies performed with small forceps was 293, and with standard forceps was 212. Although the proportion of sample specimens that contained tumor cells per biopsy with each forceps was comparable (small forceps biopsy: 73%, standard forceps biopsy; 71%. *p* = 0.75), the median tumor size obtained with a standard forceps biopsy (0.16 mm^2^) was larger than that obtained with a small forceps biopsy (0.12 mm^2^) (*p* < 0.01) (Figure S2). Third, we evaluated the change of tumor size with each successive biopsy in group C due to low tissue and tumor size in the group, and it did not show a decrease in subsequent biopsy samples (Figure S3).

## DISCUSSION

To our knowledge, this is the first report to evaluate the analytical performance of the ODxTT on EBB/TBB samples divided into three groups of different biopsy forceps size: only standard forceps use, only small forceps use, and using both size forceps, in clinical settings. Our results showed that the TBB cases performed using only small forceps were prone to unsuccessful analysis. The reason for the lower success rate of ODxTT in this group, compared with standard forceps biopsy, was due to an insufficient amount of nucleic acid, and not due to a low quality of nucleic acids. A retrospective study reported a favorable success rate for the ODxTT when tumor specimens with a tissue size of 4 mm^2^ or larger were used.^7^ Although the tissue sizes between the groups containing the standard forceps biopsy samples (combined groups A and B) and the group containing only small forceps biopsy samples (group C) was not significantly different, the proportion with tissue sizes 4 mm^2^ or larger was significantly lower in group C compared with combined groups A and B. Furthermore, the tumor sizes were also significantly smaller in group C compared with combined groups A and B in the exploratory analysis.

Considering the findings in our report, to improve the success rate of the ODxTT for the cases using an MP290F scope or thin bronchoscope with small EBUS‐GS method, an additional strategy is needed to obtain a sufficient amount of tissue. One simple strategy is to increase the number of samplings. Although a prospective study showed that the quantity of tumor cells from subsequent biopsies decreased, which might be due to localized bleeding resulting from repeated biopsies,^10^ our exploratory analysis did not show a decrease in tumor area with each successive biopsy in group C. We should consider changing the sampling position, that is, proximal, central, or distal within the lesion, or consider changing the angle of the bronchoscope to be as close to the lesion as possible in order to avoid the possibility of a biopsy blood clot resulting from performing the biopsy in same position. Another strategy is increasing the amount of tumor tissue taken in one sample. Our exploratory analysis suggests that tumor size in one sample obtained with TBB using standard forceps without GS is larger than that obtained with a small EBUS‐GS TBB. It has previously been reported that adding a TBB using standard forceps without GS following a small EBUS‐GS TBB is a useful procedure for improving diagnostic yield.^11^, ^12^ Although, whether conducting an EBUS‐TBB with or without a GS would result in a better diagnostic yield is controversial. The latest large randomized study comparing the EBUS‐TBB, both with and without GS for small peripheral pulmonary lesions, showed that the diagnostic yield of EBUS‐TBB with a GS was significantly higher than that without a GS.^13^ Another retrospective study showed that the diagnostic yield of TBB using EBUS‐GS for small peripheral pulmonary lesions reached a plateau at the fifth biopsy.^14^ Therefore, when using a thin bronchoscope for peripheral pulmonary lesions, it is considered a useful strategy to first perform a small EBUS‐GS TBB about five times for diagnosis, then additionally perform a TBB using standard forceps in cases where we can proceed with the tip of scope close to the lesion, and reproducibly identify areas within the lesions using EBUS without a GS. This is the method used in group B in our strategy for obtaining sufficient sample volume. This strategy improved both the success rate of the ODxTT and tissue size compared with the group containing only small forceps biopsy samples. It is unknown about the usefulness of the strategy of additional TBB using standard forceps without GS solely relying on fluoroscopy images after small EBUS‐GS TBB when we cannot reproducibly identify areas within the lesions using EBUS without a GS. Cryobiopsy could also be a useful option for obtaining larger specimens compared with forceps biopsy.^15^, ^16^ In recent years, a novel 1.1 mm cryoprobe, which can pass through the 1.7 mm working channel of a BF‐MP 290F, became available in clinical settings, and therefore it is expected that large specimens can even be collected with an ultrathin bronchoscope.^17^ It is also important to consider trying another sampling method, such as EBUS‐TBNA, CTNB and surgical biopsy, if the specimen obtained from EBB/TBB is not appropriate for NGS analysis.^7^, ^8^, ^10^

There were several limitations to this study. First, this study was a small retrospective study, therefore, further evaluation with a larger cohort is required. Second, this study was conducted in a single institution, and the results of this study may not be applicable to other institutions because the strategy of tissue sampling methods, the sample preparation process, and the judgment of whether or not to submit a sample for ODxTT vary in each institution. Third, although tumor cell content is one of the important factors for ODxTT analysis, we did not record which exact specimens were submitted among all specimens taken for a given patient in many cases. We could not, therefore, evaluate the relationship between tumor cell content and the result of the ODxTT. Finally, we could not evaluate the submission rate of ODxTT among the groups because the decision to submit for ODxTT was based on a variety of factors other than the amount of tumor cells and tumor content, including the extent of necrosis, clinical stage, ease of rebiospy, and the urgency of anticancer drug treatment.

In conclusion, the analysis of ODxTT for TBB specimens using only small forceps is prone to be unsuccessful due to an insufficient amount of nucleic acid. Therefore, an additional supplemental strategy is needed to address the problems for the analysis of ODxTT when TBB using an ultrathin bronchoscope, or small EBUS‐GS TBB using a thin bronchoscope, is performed.

## CONFLICT OF INTEREST

Matsusaka Municipal Hospital received a research grant funding from Novartis, GlaxoSmithKline, AstraZeneca, Daiichi Sankyo, Bayer, and Boehringer Ingelheim. K. Ito has received speaker fees as honoraria from Eli Lilly Japan, Chugai, AstraZeneca, MSD, Boehringer Ingelheim Japan, Ono, and Pfizer Japan. O. Taguchi received speaker fees as honoraria from AstraZeneca. O. Hataji received speaker fees as honoraria from Novartis Pharma, AstraZeneca, and Boehringer Ingelheim Japan. The remaining authors declare no conflict of interest.

## Supporting information


Appendix
