# Supplementary material for: Comparison of the analytical performance of the Oncomine dx target test focusing on bronchoscopic biopsy forceps size in non‐small cell lung cancer
Source: Thorac Cancer. 2022 Apr 5;13(10):1449–56. doi: 10.1111/1759-7714.14411 (PMC9108074; doi:10.1111/1759-7714.14411)
Supplement: Supplementary file 1 — Appendix [file TCA-13-1449-s001.docx]

**Supplemental Figures**

**
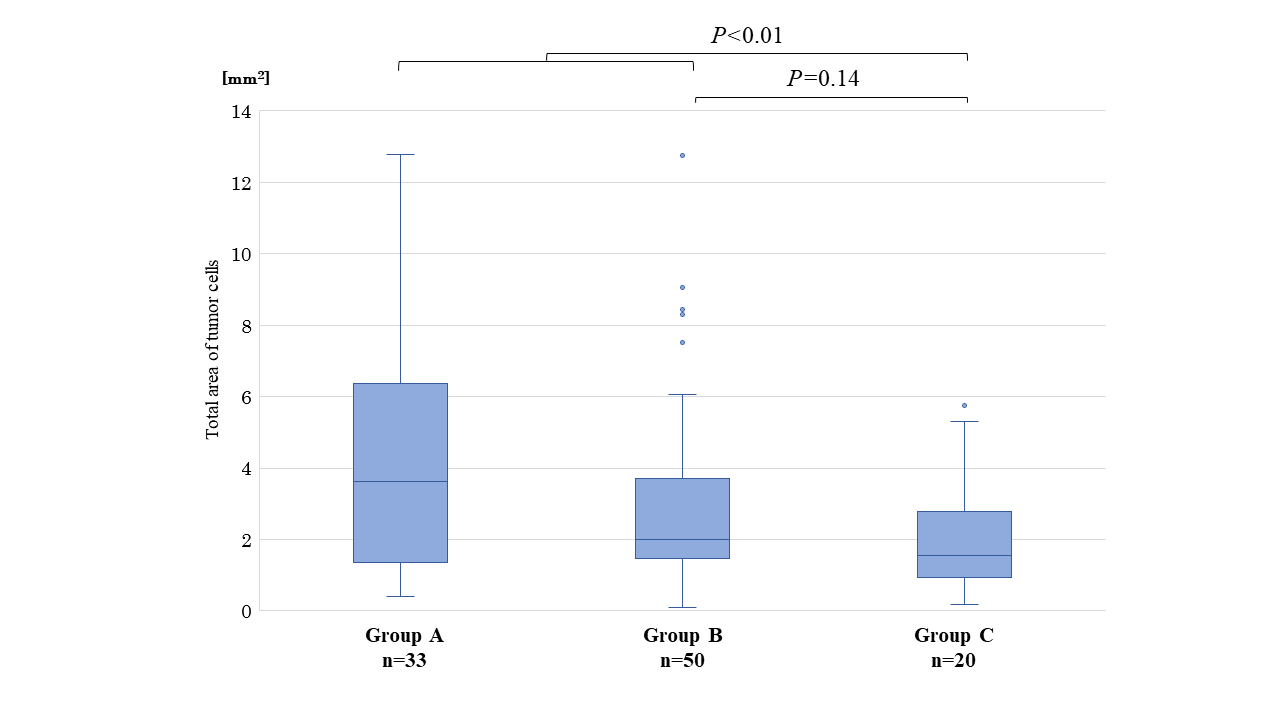
**

**Supplemental Fig. 1.** **The comparison of tumor size**

The tumor size was evaluated as the sum of tumor areas for each case. P-values of less than 0.05 were considered statistically significant.


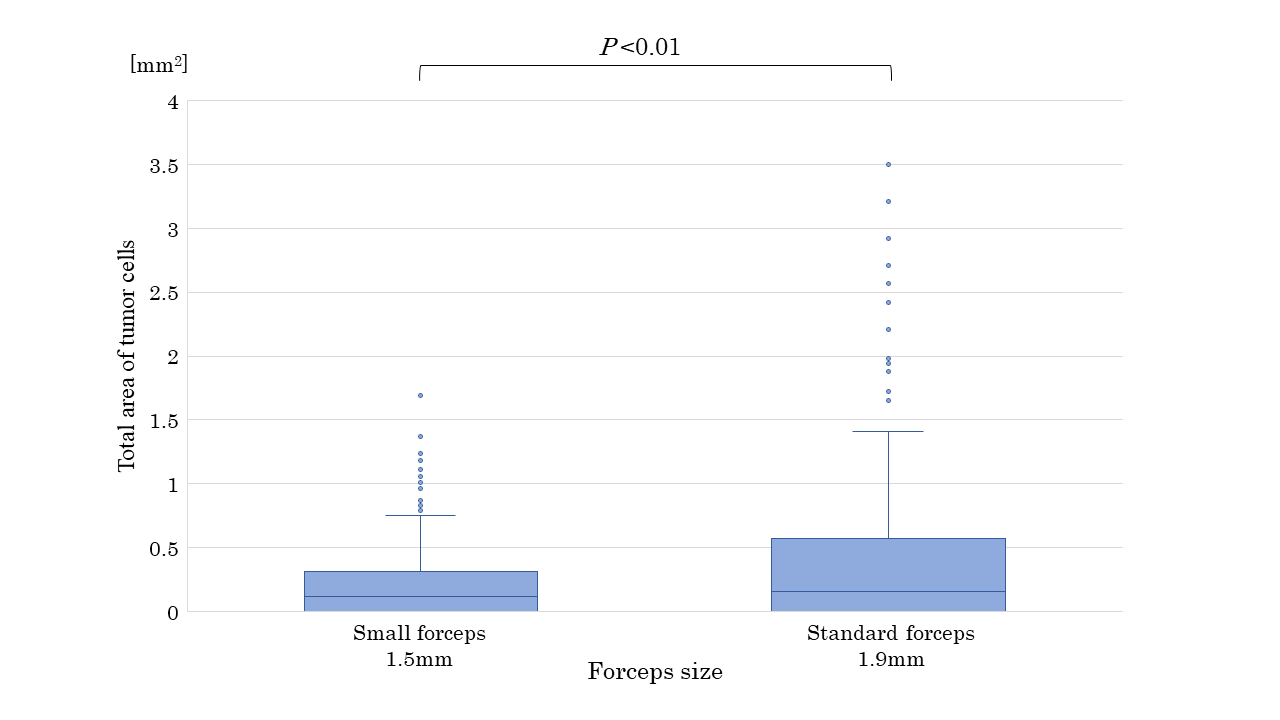


**Supplemental Fig. 2. Tumor sizes in one sample obtained by small forceps biopsy and standard forceps biopsy**.


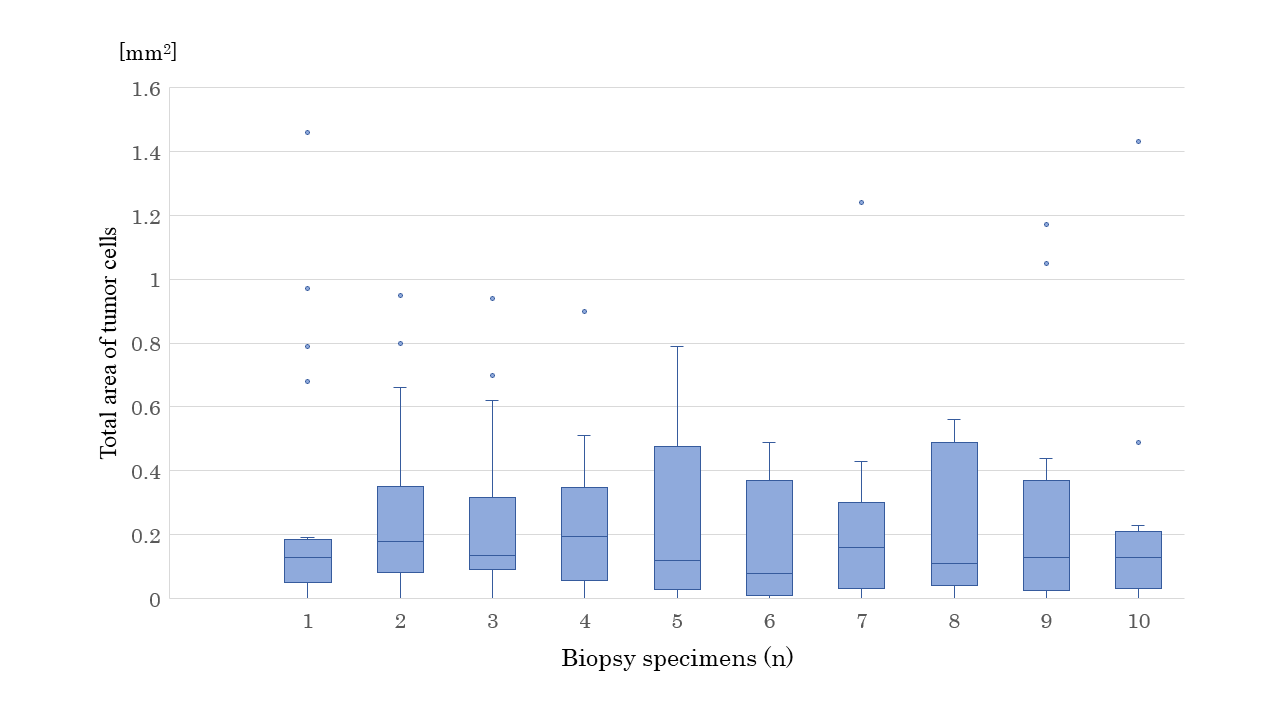


**Supplemental Fig. 3.** **Tumor sizes in each successive biopsy for the group containing only small forceps biopsy samples.**

Median tumor sizes with interquartile range in each successive biopsy were shown.
